# RCSB protein Data Bank: exploring protein 3D similarities via comprehensive structural alignments

**DOI:** 10.1093/bioinformatics/btae370

**Published:** 2024-06-13

**Authors:** Sebastian Bittrich, Joan Segura, Jose M Duarte, Stephen K Burley, Yana Rose

**Affiliations:** Research Collaboratory for Structural Bioinformatics Protein Data Bank, San Diego Supercomputer Center, University of California, La Jolla, CA 92093, United States; Research Collaboratory for Structural Bioinformatics Protein Data Bank, San Diego Supercomputer Center, University of California, La Jolla, CA 92093, United States; Research Collaboratory for Structural Bioinformatics Protein Data Bank, San Diego Supercomputer Center, University of California, La Jolla, CA 92093, United States; Research Collaboratory for Structural Bioinformatics Protein Data Bank, San Diego Supercomputer Center, University of California, La Jolla, CA 92093, United States; Research Collaboratory for Structural Bioinformatics Protein Data Bank, Rutgers, The State University of New Jersey, Piscataway, NJ 08854, United States; Institute for Quantitative Biomedicine, Rutgers, The State University of New Jersey, Piscataway, NJ 08854, United States; Department of Chemistry and Chemical Biology, Rutgers, The State University of New Jersey, Piscataway, NJ 08854, United States; Cancer Institute of New Jersey, Rutgers, The State University of New Jersey, New Brunswick, NJ 08901, United States; Research Collaboratory for Structural Bioinformatics Protein Data Bank, San Diego Supercomputer Center, University of California, La Jolla, CA 92093, United States

## Abstract

**Motivation:**

Tools for pairwise alignments between 3D structures of proteins are of fundamental importance for structural biology and bioinformatics, enabling visual exploration of evolutionary and functional relationships. However, the absence of a user-friendly, browser-based tool for creating alignments and visualizing them at both 1D sequence and 3D structural levels makes this process unnecessarily cumbersome.

**Results:**

We introduce a novel pairwise structure alignment tool (rcsb.org/alignment) that seamlessly integrates into the RCSB Protein Data Bank (RCSB PDB) research-focused RCSB.org web portal. Our tool and its underlying application programming interface (alignment.rcsb.org) empowers users to align several protein chains with a reference structure by providing access to established alignment algorithms (FATCAT, CE, TM-align, or Smith–Waterman 3D). The user-friendly interface simplifies parameter setup and input selection. Within seconds, our tool enables visualization of results in both sequence (1D) and structural (3D) perspectives through the RCSB PDB RCSB.org Sequence Annotations viewer and Mol* 3D viewer, respectively. Users can effortlessly compare structures deposited in the PDB archive alongside more than a million incorporated Computed Structure Models coming from the ModelArchive and AlphaFold DB. Moreover, this tool can be used to align custom structure data by providing a link/URL or uploading atomic coordinate files directly. Importantly, alignment results can be bookmarked and shared with collaborators. By bridging the gap between 1D sequence and 3D structures of proteins, our tool facilitates deeper understanding of complex evolutionary relationships among proteins through comprehensive sequence and structural analyses.

**Availability and implementation:**

The alignment tool is part of the RCSB PDB research-focused RCSB.org web portal and available at rcsb.org/alignment. Programmatic access is available via alignment.rcsb.org. Frontend code has been published at github.com/rcsb/rcsb-pecos-app. Visualization is powered by the open-source Mol* viewer (github.com/molstar/molstar and github.com/molstar/rcsb-molstar) plus the Sequence Annotations in 3D Viewer (github.com/rcsb/rcsb-saguaro-3d).

## 1 Introduction

Pairwise alignments of 3D structures of proteins are invaluable for understanding evolutionary relationships between and among proteins, to infer functional annotations from well-studied protein structures, and for analyses of Computed Structure Models (CSMs), e.g. from AlphaFold DB (Varadi 2024), ModelArchive (Schwede 2009), or ESM Metagenomic Atlas (Lin 2023). Moreover, the success of machine learning-based protein structure prediction underlines the relevance of these tools as they can be used to transfer ligand binding information from experimentally-determined structures to CSMs or align multiple individual experimentally-determined structures of domains to the CSM of the full-length polypeptide chain.

In this work, we present the RCSB Protein Data Bank (RCSB PDB) research-focused RCSB.org alignment tool, which provides an intuitive graphical user interface (rcsb.org/alignment) plus an application programming interface (API, alignment.rcsb.org). These entry points empower users to align multiple 3D structures with one reference structure in a pairwise manner. These tools succeed a previously provided RCSB.org structure alignment capabilities (Prlić 2010). Uniquely, our new tool distinguishes itself from comparable solutions (Li 2014, Wiederstein and Sippl 2020, Holm et al. 2023, van Kempen 2023, Prochazka 2024, Zurkowski 2024) by offering a powerful range of parameterizable alignment methods. Users can explore these alignments, both at the 1D sequence level and in 3D, ensuring a seamless and comprehensive experience.

## 2 Results

The RCSB.org alignment tool (rcsb.org/alignment) facilitates 3D superposition of multiple protein structures to one reference structure and integrates with other RCSB.org web portal APIs (Rose 2021, Burley 2022, 2023, Bittrich 2023).

### 2.1 Required input and options

Individual structures can be selected for alignment by typing in the corresponding PDB entry identifier, by pasting a URL to a file [mmCIF, BinaryCIF (Sehnal 2020], or PDB formats are all supported), or by uploading an externally-provided atomic coordinates file (in various file formats, see Section 2.2). One polypeptide chain must be selected for each entry. Optionally, a sequence range can be included to align only a specific set of residues (and ignore the others). For the alignment, users can choose from a range of established methods, including jFATCAT, jCE, jCE-CP, Smith–Waterman 3D (Lafita 2019), and TM-align (Zhang and Skolnick 2005). Algorithm-specific parameters can also be specified. jFATCAT supports both rigid and flexible alignments: rigid alignments will optimize the superposition of two protein chains in their entirety, whereas flexible alignments can fragment chains into smaller sections and align fragment pairs, while allowing for flexibility in regions connecting fragments. Flexible alignments are particularly useful for partially disordered proteins or those with flexible hinges such as calmodulin or the catalytic domains of protein kinases.

### 2.2 File upload and URL support

Users can align structures from arbitrary URLs in any of the supported formats: mmCIF, BinaryCIF (Sehnal 2020), plus the legacy PDB format.

Local files can serve as input by selecting the “File Upload” input mode. Files are processed by a dedicated API at user-upload.rcsb.org. The API endpoint responds to file uploads with a unique token, which can be used to retrieve the file and initially restricts file access to the original uploader but can be shared freely. The access token grants access to the file for 90 days. Uploaded files can be referenced by their URLs.

### 2.3 Result overview

**Figure 1.**
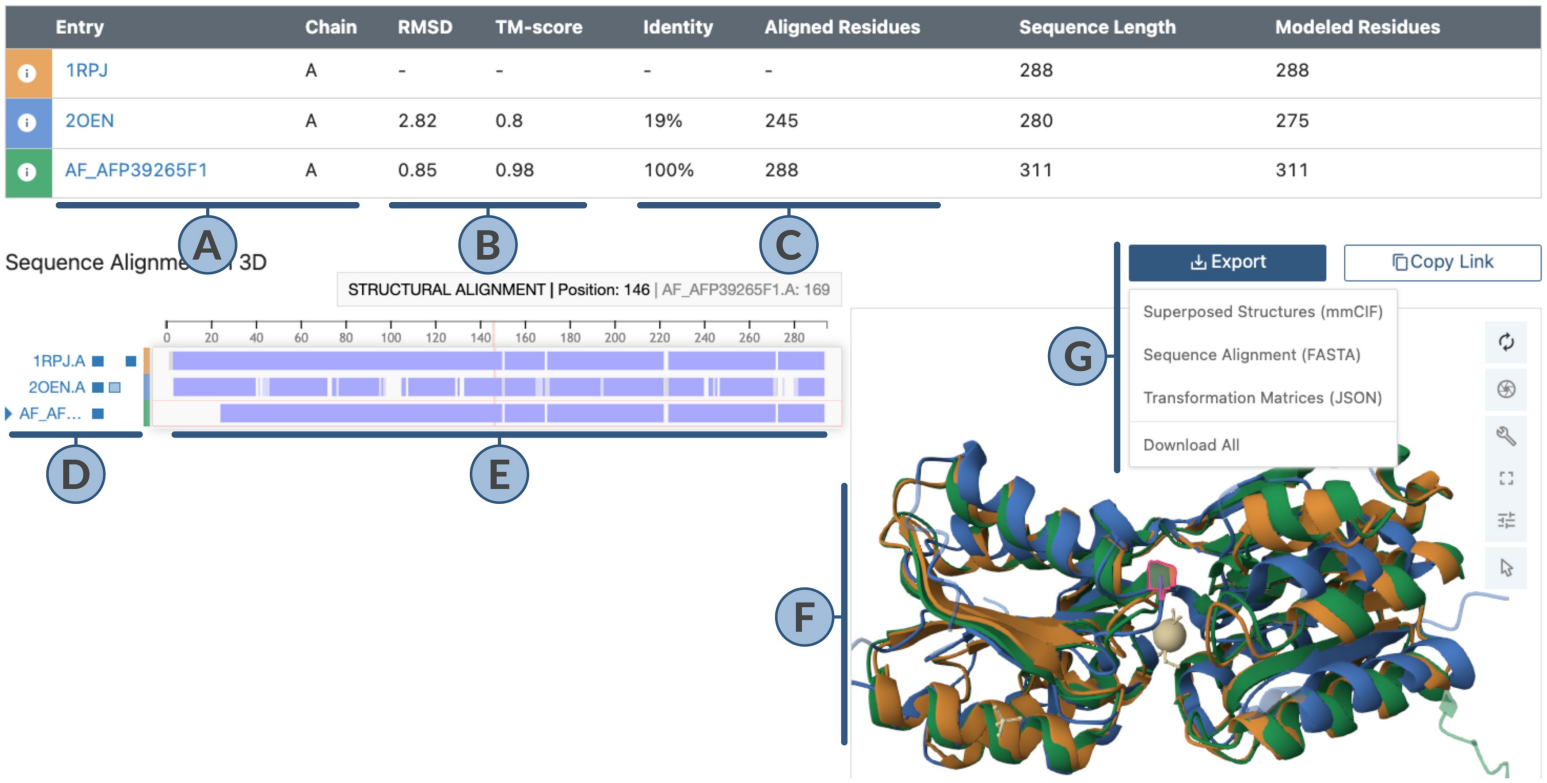
Output of the pairwise alignment application. (A) Summary of aligned chains. (B) 3D Structure-based scores (Root-Mean-Square-Deviation or RMSD in Ångstrom Units; TM-score, range 0.00–1.00). (C) Sequence-based scores (% identity, number of aligned residues). (D) Toggle visibility of aligned chain, all polymers, and all ligands via 1st, 2nd, and 3rd box buttons, respectively. (E) Structurally aligned sequence regions in a darker shade, regions aligned only at sequence level in a lighter shade, non-aligned gaps are left empty. (F) 3D visualization of aligned structures. (G) Tools to export and share alignment.

Figure 1 showcases the output of a structure alignment. Chain A of 1rpj (D-allose binding protein) serves as reference, chain A of 2oen (distant, sugar-binding homolog) and chain A of AF-P39265-F1 [CSM of D-allose binding protein with UniProt identifier P39265, (Varadi 2024)] were aligned to this reference structure.

For all input structures (Fig. 1A) except the first listed or reference structure, scores of their 3D structure (Fig. 1B) and 1D sequence alignment (Fig. 1C) with respect to the reference are reported. An interactive combination of a 1D sequence view (bottom-left panel) and 3D structure view (bottom-right panel) allows users to interrogate the alignment. The blue boxes on the left side of the sequence tracks (Fig. 1D) summarize the content of each aligned structure by providing a break-down of the aligned polymer chain (1st box), other polymer chains (2nd box), and non-polymer ligands (3rd box). These boxes can be used to toggle the representation of the corresponding item. Hovering over 1D sequence positions (Fig. 1E) or amino acid residues in 3D (Fig. 1F) will highlight the corresponding residue(s), both at the 1D sequence level and in 3D. Sequence features are represented by the RCSB Sequence Annotations Viewer (Segura 2020, 2022). 3D visualization is powered by the Mol* 3D viewer (Sehnal 2021).

### 2.4 Exporting and sharing alignments

Several result files are offered for download (Fig. 1G): Transformed mmCIF files can be reopened in a 3D viewer that will show all structures superimposed, sequence alignments are available as FASTA format file, and 3D transformation matrices are offered in JSON format.

Alignment results can be bookmarked and shared with other users by obtaining a URL that includes all inputs and parameters needed to recreate an alignment.

### 2.5 Structure alignment API

The Pairwise Structure Alignment tool is built atop a public API (alignment.rcsb.org), which provides a convenient way to compute structure alignments in a pairwise manner. Time required for computation depends on structure size and the alignment method used. Because some comparisons can take considerable time, alignment jobs run asynchronously. Each user request is assigned a unique identifier in the form of a ticket. This ticket serves as a key to track the progress of the alignment job. Users can check the status of their ticket, allowing them to monitor the processing stages until the job reaches completion.

## 3 Conclusion

The RCSB PDB research-focused RCSB.org web portal Pairwise Structure Alignment application is a novel tool that allows users to align multiple 3D protein structures to one reference protein chain. Selection from a number of well-established alignment methods is permitted and can be parameterized freely. Alignments of experimentally-determined structures from the PDB archive, CSMs from AlphaFold DB and the ModelArchive are integrated, and data from public URLs plus uploading of user-supplied atomic coordinates files are all supported. Moreover, structure data can be referenced using UniProt accession numbers, AlphaFold DB identifiers, or ESM Metagenomic Atlas identifiers. A combination of the RCSB Sequence Annotations Viewer (Segura 2020, 2022) and the Mol* 3D viewer (Sehnal 2021) enables interactive interrogation of alignments by users, both at the 1D sequence level and in 3D. Resulting alignments can be saved in different formats and can be bookmarked and shared via URL.

## References

[btae370-B1] Bittrich S , Bhikadiya C, Bi C et al RCSB protein data bank: efficient searching and simultaneous access to one million computed structure models alongside the PDB structures enabled by architectural advances. J Mol Biol 2023;435:167994. 36738985 10.1016/j.jmb.2023.167994 PMC11514064

[btae370-B2] Burley SK , Bhikadiya C, Bi C et al RCSB protein data bank: tools for visualizing and understanding biological macromolecules in 3D. Protein Sci 2022;31:e4482. 36281733 10.1002/pro.4482 PMC9667899

[btae370-B3] Burley SK , Bhikadiya C, Bi C et al RCSB protein data bank (RCSB. org): delivery of experimentally-determined PDB structures alongside one million computed structure models of proteins from artificial intelligence/machine learning. Nucleic Acids Res 2023;51:D488–508. 36420884 10.1093/nar/gkac1077 PMC9825554

[btae370-B4] Holm L , Laiho A, Törönen P et al DALI shines a light on remote homologs: one hundred discoveries. Protein Sci 2023;32:e4519. 36419248 10.1002/pro.4519 PMC9793968

[btae370-B5] Lafita A , Bliven S, Prlić A et al BioJava 5: a community driven open-source bioinformatics library. PLoS Comput Biol 2019;15:e1006791. 30735498 10.1371/journal.pcbi.1006791 PMC6383946

[btae370-B6] Li Z , Natarajan P, Ye Y et al POSA: a user-driven, interactive multiple protein structure alignment server. Nucleic Acids Res 2014;42:W240–5. 24838569 10.1093/nar/gku394 PMC4086100

[btae370-B7] Lin Z , Akin H, Rao R et al Evolutionary-scale prediction of atomic-level protein structure with a language model. Science 2023;379:1123–30. 36927031 10.1126/science.ade2574

[btae370-B8] Prlić A , Bliven S, Rose PW et al Pre-calculated protein structure alignments at the RCSB PDB website. Bioinformatics 2010;26:2983–5. 20937596 10.1093/bioinformatics/btq572 PMC3003546

[btae370-B9] Prochazka D , Slaninakova T, Olha J et al AlphaFind: discover structure similarity across the proteome in AlphaFold DB. *Nucleic Acids Res* 2024;gkae397 . 10.1093/nar/gkae397 PMC11223785 38747341

[btae370-B10] Rose Y , Duarte JM, Lowe R et al RCSB protein data bank: architectural advances towards integrated searching and efficient access to macromolecular structure data from the PDB archive. J Mol Biol 2021;433:166704. 33186584 10.1016/j.jmb.2020.11.003 PMC9093041

[btae370-B11] Schwede T , Sali A, Honig B et al Outcome of a workshop on applications of protein models in biomedical research. Structure 2009;17:151–9. 19217386 10.1016/j.str.2008.12.014 PMC2739730

[btae370-B12] Segura J , Rose Y, Westbrook J et al RCSB protein data bank 1D tools and services. Bioinformatics 2020;36:5526–7. 10.1093/bioinformatics/btaa1012 PMC8016458 33313665

[btae370-B13] Segura J , Rose Y, Bittrich S et al RCSB protein data bank 1D3D module: displaying positional features on macromolecular assemblies. Bioinformatics 2022;38:3304–5. 35543462 10.1093/bioinformatics/btac317 PMC9191206

[btae370-B14] Sehnal D , Bittrich S, Velankar S et al BinaryCIF and CIFTools—lightweight, efficient and extensible macromolecular data management. PLoS Comput Biol 2020;16:e1008247. 33075050 10.1371/journal.pcbi.1008247 PMC7595629

[btae370-B15] Sehnal D , Bittrich S, Deshpande M et al Mol* viewer: modern web app for 3d visualization and analysis of large biomolecular structures. Nucleic Acids Res 2021;49:W431–7. 33956157 10.1093/nar/gkab314 PMC8262734

[btae370-B16] van Kempen M , Kim SS, Tumescheit C et al Fast and accurate protein structure search with foldseek. Nat Biotechnol 2023;42:243–6. 37156916 10.1038/s41587-023-01773-0 PMC10869269

[btae370-B17] Varadi M , Bertoni D, Magana P et al AlphaFold protein structure database in 2024: providing structure coverage for over 214 million protein sequences. Nucleic Acids Res 2024;52:D368–75. 37933859 10.1093/nar/gkad1011 PMC10767828

[btae370-B18] Wiederstein M , Sippl MJ. TopMatch-web: pairwise matching of large assemblies of protein and nucleic acid chains in 3D. Nucleic Acids Res 2020;48:W31–5. 32479639 10.1093/nar/gkaa366 PMC7319569

[btae370-B19] Zhang Y , Skolnick J. TM-align: a protein structure alignment algorithm based on the TM-score. Nucleic Acids Res 2005;33:2302–9. 15849316 10.1093/nar/gki524 PMC1084323

[btae370-B20] Zurkowski M , Swiercz M, Wozny F et al RNAhugs web server for customized 3D RNA structure alignment. Nucleic Acids Res 2024;gkae259. 38587206 10.1093/nar/gkae259 PMC11223877

